# Supplementary material for: Developing a general AI model for integrating diverse genomic modalities and comprehensive genomic knowledge
Source: bioRxiv. 2025 May 14:2025.05.08.652986. Preprint. [Version 1] doi: 10.1101/2025.05.08.652986 (PMC12132192; doi:10.1101/2025.05.08.652986)
Supplement: Supplement 1 [file NIHPP2025.05.08.652986v1-supplement-1.pdf]

bioRxiv preprint doi: https://doi.org/10.1101/2025.05.08.652986; this version posted May 14, 2025. The copyright holder for this preprint (which was not certified by peer review) is the author/funder, who has granted bioRxiv a license to display the preprint in perpetuity. It is made available under aCC-BY 4.0 International license.

Supplementary information

Data processing

All sequencing data used in our model is in the hg38 version for humans and the mm10 version for mice. Since our model utilizes sequencing data from diverse cells and tissues in the training, we aimed to remove distribution shifts during training. For the TF and histone mark ChIP-seq data, we utilized their signal p-values and performed z-score normalization. We then clipped the normalized signal values within the range (-2, 36) to exclude extreme values. For all expression data, including CAGE-seq, RNA-seq, Bru-seq, BruUV-seq, BruChase-seq, TT-seq, GRO-seq, GRO-cap, PRO-seq, and Net-CAGE, we followed the normalization method used in GraphReg [92] for cross-cell type prediction. We applied RPGC normalization [93] to generate the bigWig files with the binsize set to the resolution to be predicted: `bamCoverage --normalizeUsing RPGC --effectiveGenomeSize 2913022398 --binSize 1000`. Subsequently, we divided each signal value by the 95th percentile of non-zero values and performed an arcsinh transformation to preserve the original distribution. This approach avoids the distributional distortions that direct logarithmic or arcsinh transformations might introduce.

For Hi-C, ChIA-PET, and Micro-C contact maps, we used Juicebox [94] to derive the KR or O/E normalized contact maps. For KR normalization, we used Juicebox straw's 'SCALE' normalization. The intact Hi-C and Micro-C contact maps at a 1kb resolution were log2 transformed and clipped within the range (-2, 10). Subsequently, the intact Hi-C data was processed using a Gaussian smoothing filter with a length of 5 and a sigma of 1, while the Micro-C data was smoothed with the same filter length but a sigma of 0.8. For the ChIA-PET data at a 5kb resolution, we applied a log2 transformation with a pseudocount of 1, followed by Gaussian smoothing with a filter length of 5 and a sigma of 1.

The ChIP-seq, CAGE-seq, RNA-seq, Bru-seq, BruUV-seq, BruChase-seq, GRO-cap, STARR-seq, and Intact Hi-C data were downloaded from ENCODE [23], while ChIA-PET and Micro-C data were sourced from 4DN. For data originating from different studies, GRO-seq was aligned using Bowtie2 [95], and both TT-seq and NET-CAGE were aligned using STAR [96].

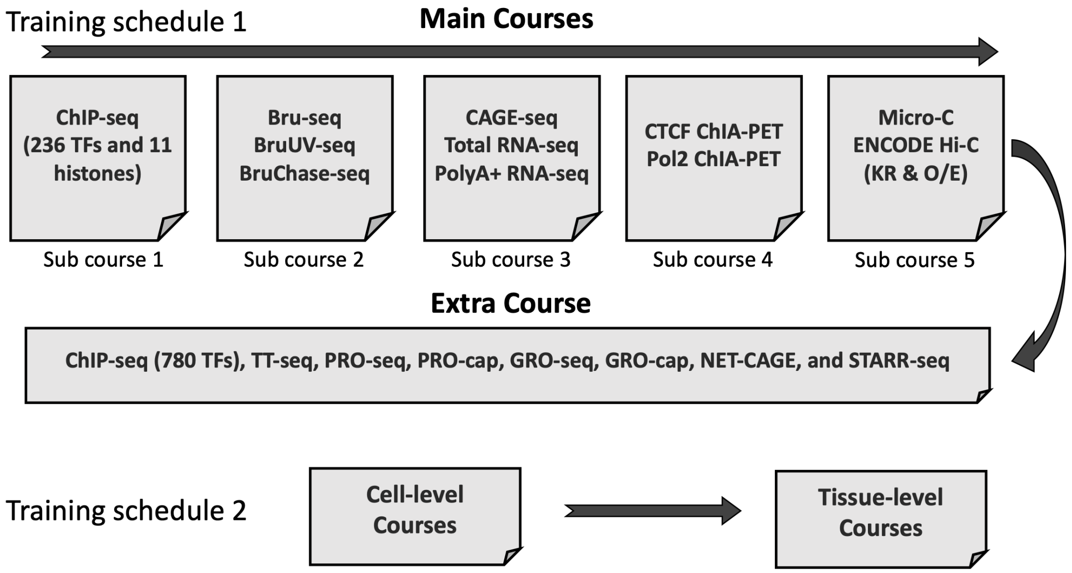

Fig. S1: Two training schedules included in our general model's curriculum learning framework. One schedule introduce the model from main courses to extra courses, and the other one introduce the model from cell data to tissue data.

bioRxiv preprint doi: https://doi.org/10.1101/2025.05.08.652986; this version posted May 14, 2025. The copyright holder for this preprint (which was not certified by peer review) is the author/funder, who has granted bioRxiv a license to display the preprint in perpetuity. It is made available under aCC-BY 4.0 International license.

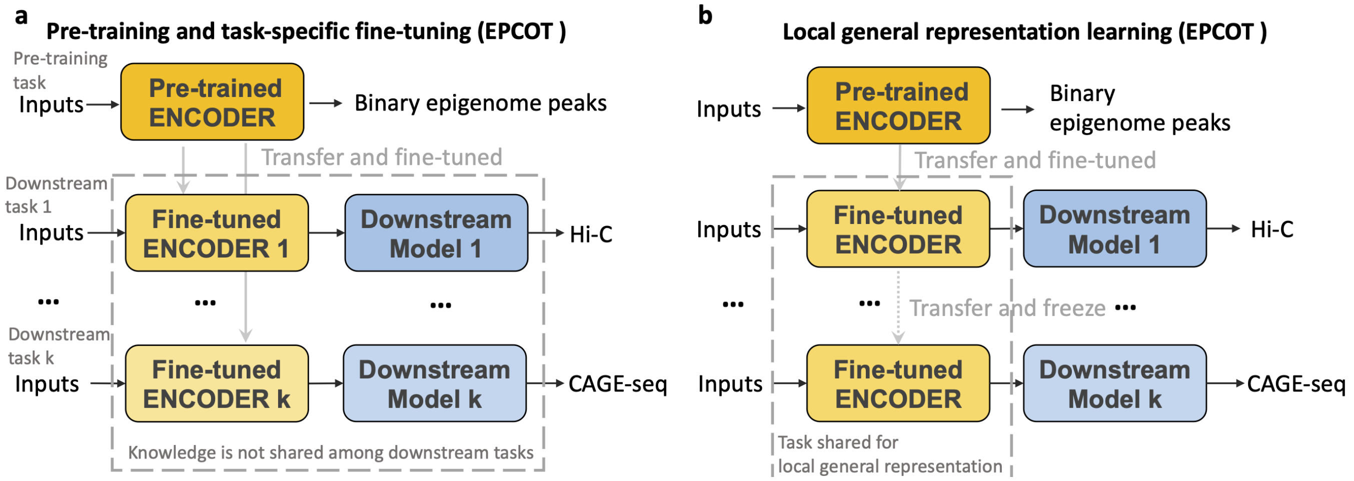

Fig. S2: Strategies used in EPCOT model. **a**, EPCOT uses a pre-training and fine-tuning framework which is pre-trainined to predict binary peaks of epigenomic features, and then the encoder is separately transferred and fine-tuned in several downstream tasks and task-specific downstream models are trained. **b**, A simple strategy used in EPCOT for general representation learning. The pre-trained encoder in epigenomic feature task is further fine-tuned in Hi-C prediction task. Then this encoder is frozen to generate local general representations for other predictive tasks.

Cell Type	Biological Process	Fold Enrichment	FDR
HC	neurofilament bundle assembly (GO:0033693)	78.69	2.52E-02
	vestibular receptor cell differentiation (GO:0060114)	78.69	2.49E-02
	intermediate filament polymerization or depolymerization (GO:0045105)	78.69	2.49E-02
	hair cell differentiation (GO:0035315)	29.95	1.02E-16
Endo	endothelial tube morphogenesis (GO:0061154)	27.02	8.79E-03
	endothelial cell morphogenesis (GO:0001886)	25.44	1.34E-03
	positive regulation of endothelial cell chemotaxis (GO:2001028)	19.08	2.09E-02
	negative regulation of endothelial cell proliferation (GO:0001937)	18.40	2.31E-06
MES	paraxial mesoderm morphogenesis (GO:0048340)	25.45	2.83E-03
	positive regulation of mesenchymal cell proliferation (GO:0002053)	18.91	4.11E-05
	paraxial mesoderm development (GO:0048339)	18.38	2.18E-03
	regulation of mesenchymal cell proliferation (GO:0010464)	17.54	9.73E-06
Immune	AIM2 inflammasome complex assembly (GO:0140970)	>100	2.26E-03
	negative regulation of MyD88-dependent toll-like receptor signaling pathway (GO:0034125)	>100	2.26E-03
	positive regulation of blood microparticle formation (GO:2000334)	>100	2.25E-03
	regulation of blood microparticle formation (GO:2000332)	>100	2.25E-03
PC/DC	vibrational conductance of sound to the inner ear (GO:0055127)	>100	4.64E-03
	regulation of inner ear receptor cell differentiation (GO:2000980)	57.16	1.23E-07
	regulation of inner ear auditory receptor cell differentiation (GO:0045607)	57.16	1.18E-07
	negative regulation of inner ear receptor cell differentiation (GO:2000981)	45.73	2.74E-02
Roof	inner ear morphogenesis (GO:0042472)	6.08	2.80E-02
	inner ear development (GO:0048839)	5.49	1.13E-03

Continued on next page...

bioRxiv preprint doi: https://doi.org/10.1101/2025.05.08.652986; this version posted May 14, 2025. The copyright holder for this preprint (which was not certified by peer review) is the author/funder, who has granted bioRxiv a license to display the preprint in perpetuity. It is made available under aCC-BY 4.0 International license.

Cell Type	Biological Process	Fold Enrichment	FDR
Roof	ear development (GO:0043583)	4.85	3.11E-03
	sensory perception of sound (GO:0007605)	4.85	3.84E-02

Table S2: Inner ear GO biological process analysis shew the predicted gene expressions were accurate. In the HC cell type, processes such as 'neurofilament bundle assembly' and 'vestibular receptor cell differentiation' exhibit high fold enrichment and low false discovery rate, suggesting that these processes are highly characteristic of hair cells. Similarly, for the Endo cell type, processes like "endothelial tube morphogenesis" and "endothelial cell morphogenesis" are prominently enriched, aligning with the known functions of endothelial cells. The MES cell type shows enrichment in mesoderm development and mesenchymal cell proliferation processes, while the Immune cell type is associated with pathways related to inflammasome assembly and toll-like receptor signaling. Additionally, the PC/DC and Roof cell types display significant enrichment in processes involved in inner ear receptor cell differentiation and ear development, respectively. These enrichment results provide strong evidence that the selected marker genes effectively capture the distinct biological characteristics of each cell type, thereby validating the accuracy of the model's predictions from a biological perspective.

bioRxiv preprint doi: https://doi.org/10.1101/2025.05.08.652986; this version posted May 14, 2025. The copyright holder for this preprint (which was not certified by peer review) is the author/funder, who has granted bioRxiv a license to display the preprint in perpetuity. It is made available under aCC-BY 4.0 International license.

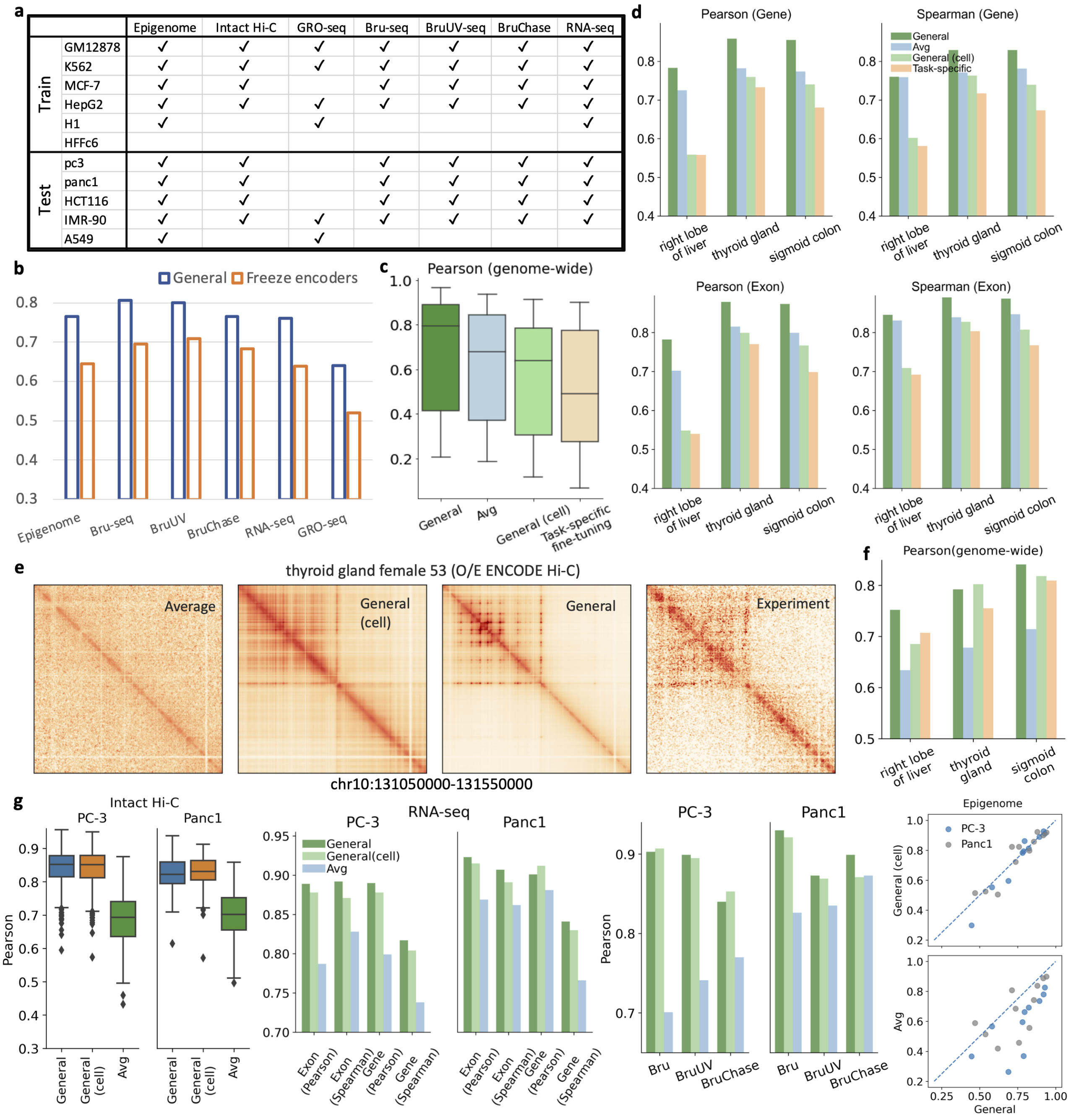

Fig. S3: See captions on the next page.

bioRxiv preprint doi: https://doi.org/10.1101/2025.05.08.652986; this version posted May 14, 2025. The copyright holder for this preprint (which was not certified by peer review) is the author/funder, who has granted bioRxiv a license to display the preprint in perpetuity. It is made available under aCC-BY 4.0 International license.

Fig. S3: Evaluation of general model's performance across diverse tasks. **a**, Availability of genomic modalities in training and testing cell lines used for evaluation. **b**, The general model outperforms the models by freezing both local and global encoders, pre-trained on epigenome prediction and then fine-tuned in Intact Hi-C prediction, which indicates better general representation learning. **c**, The general model incorporating multiple tissue data outperforms all other approaches including average signal experiment, the general model trained only on cell lines, and task-specific fine-tuning models, in predicting epigenomic features in three unseen tissues. **d,f**, The general model incorporating multiple tissue data outperforms all other approaches in the RNA-seq prediction task across genomic bins associated with the whole genome, genes, and exons in chromosomes 10 and 21. **e**, A region is illustrated to demonstrate that the general model predicts the Intact Hi-C contact map more accurately than the average Hi-C map. **g**, The general models, whether or not including multiple tissue data in the training, perform similarly across different tasks and both outperform average signal experiments.

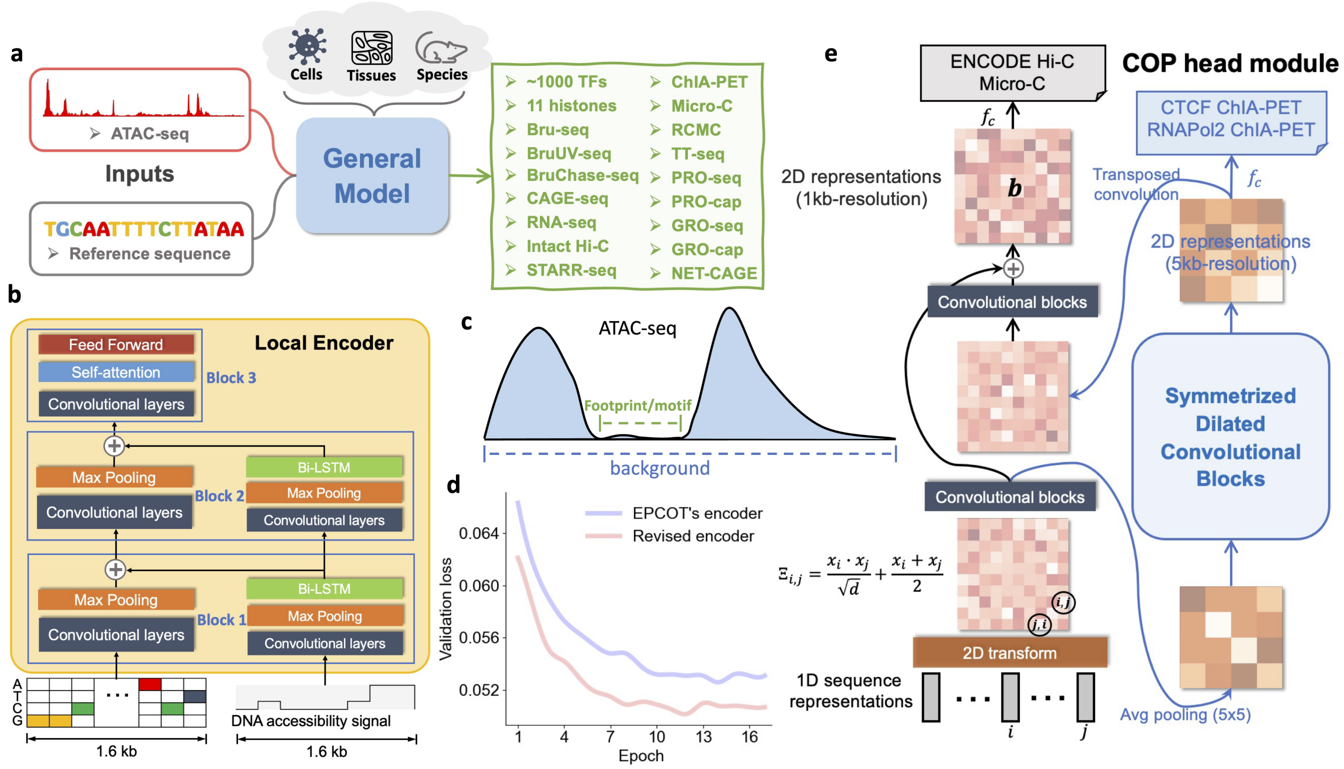

Fig. S4: Model architecture illustration of the general model. a, An overview of the proposed general model. b, The model architecture of local encoder. c, An illustration of ATAC-seq experiments. d, The revised local encoder used in the general model achieves lower validation loss comparing to the encoder used in EPCOT. e, The architecture of the COP head module is depicted. 1D sequence representations are initially converted into 2D features Ξ, each representing chromatin contacts at 1kb-resolution. These features undergo convolution and pooling, followed by dilated convolutional layers to achieve 2D representations at a 5kb resolution, which are then utilized to predict ChIA-PET interactions. The representations are subsequently upsampled to 1kb-resolution 2D features, which are further refined through additional convolutional layers. A residual connection integrates these features with the pre-pooling 2D features, ultimately yielding refined 2D representations at 1kb resolution used to predict Intact Hi-C and Micro-C data.

bioRxiv preprint doi: https://doi.org/10.1101/2025.05.08.652986; this version posted May 14, 2025. The copyright holder for this preprint (which was not certified by peer review) is the author/funder, who has granted bioRxiv a license to display the preprint in perpetuity. It is made available under aCC-BY 4.0 International license.

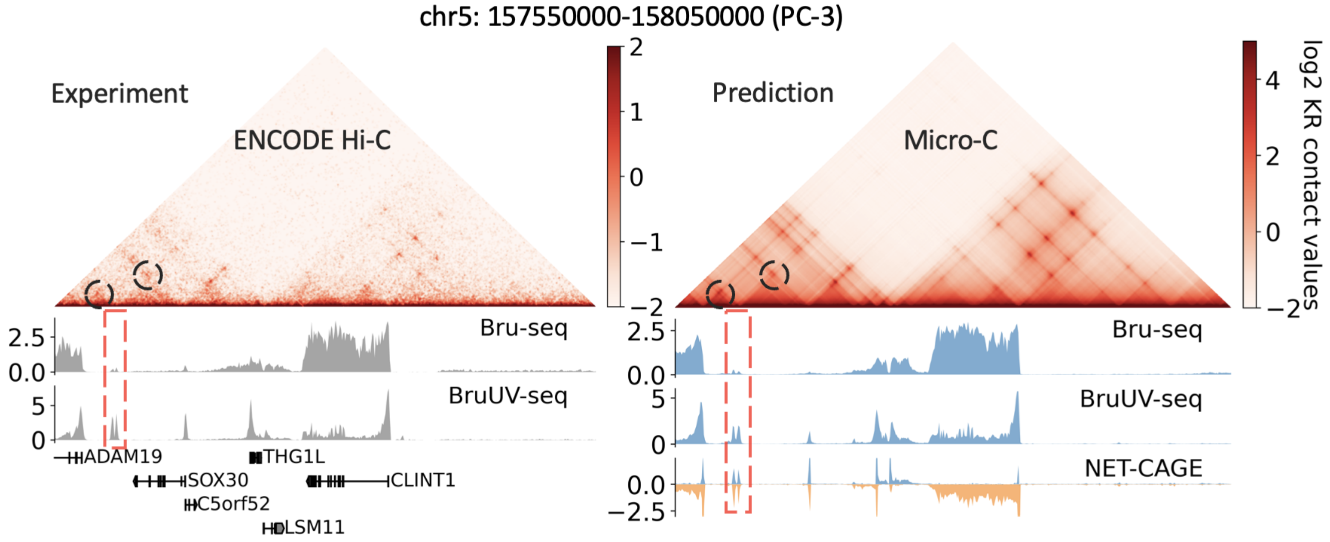

Fig. S5: A 500 kb region in the PC-3 testing cell line demonstrates the accurate prediction of putative eRNAs (highlighted in red dashed boxes) and chromatin interactions with neighboring genes ADAM19 and SOX30 (highlighted in black dashed circles). These are validated by Hi-C, Bru-seq, and BruUV-seq experiments.

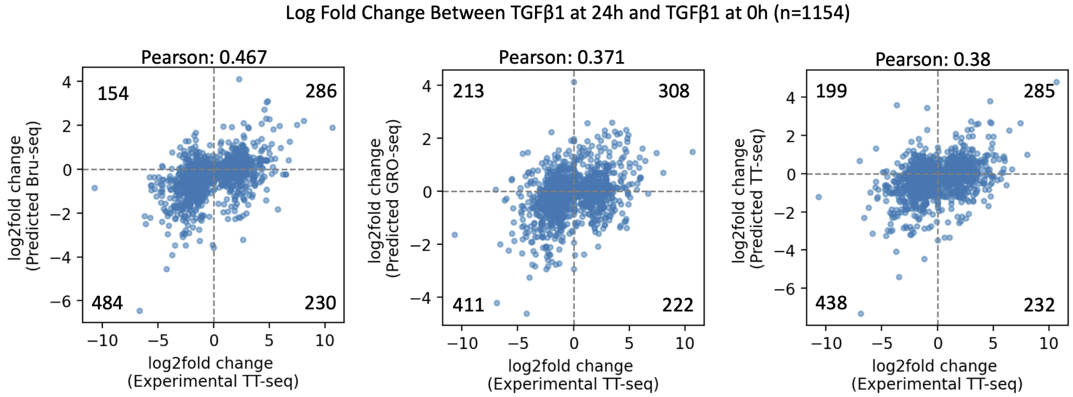

Fig. S6: Correlation of predicted and experimental log2 fold changes in nascent RNA expression between TGFβ-1 treatment at 24 hours and 0 hours across differentially expressed genes. Log fold changes predicted from Bru-seq, Gro-seq, and TT-seq are compared to the log fold changes obtained from experimental TT-seq. The results demonstrate the model's ability to capture treatment-induced changes in gene expression. Numbers in each quadrant represent the count of data points in that region, corresponding to agreement or disagreement between predicted and experimental log2 fold changes. Having more Bru-seq data included in the training likely contributed to achieving the highest correlation score for Bru-seq.

bioRxiv preprint doi: https://doi.org/10.1101/2025.05.08.652986; this version posted May 14, 2025. The copyright holder for this preprint (which was not certified by peer review) is the author/funder, who has granted bioRxiv a license to display the preprint in perpetuity. It is made available under aCC-BY 4.0 International license.

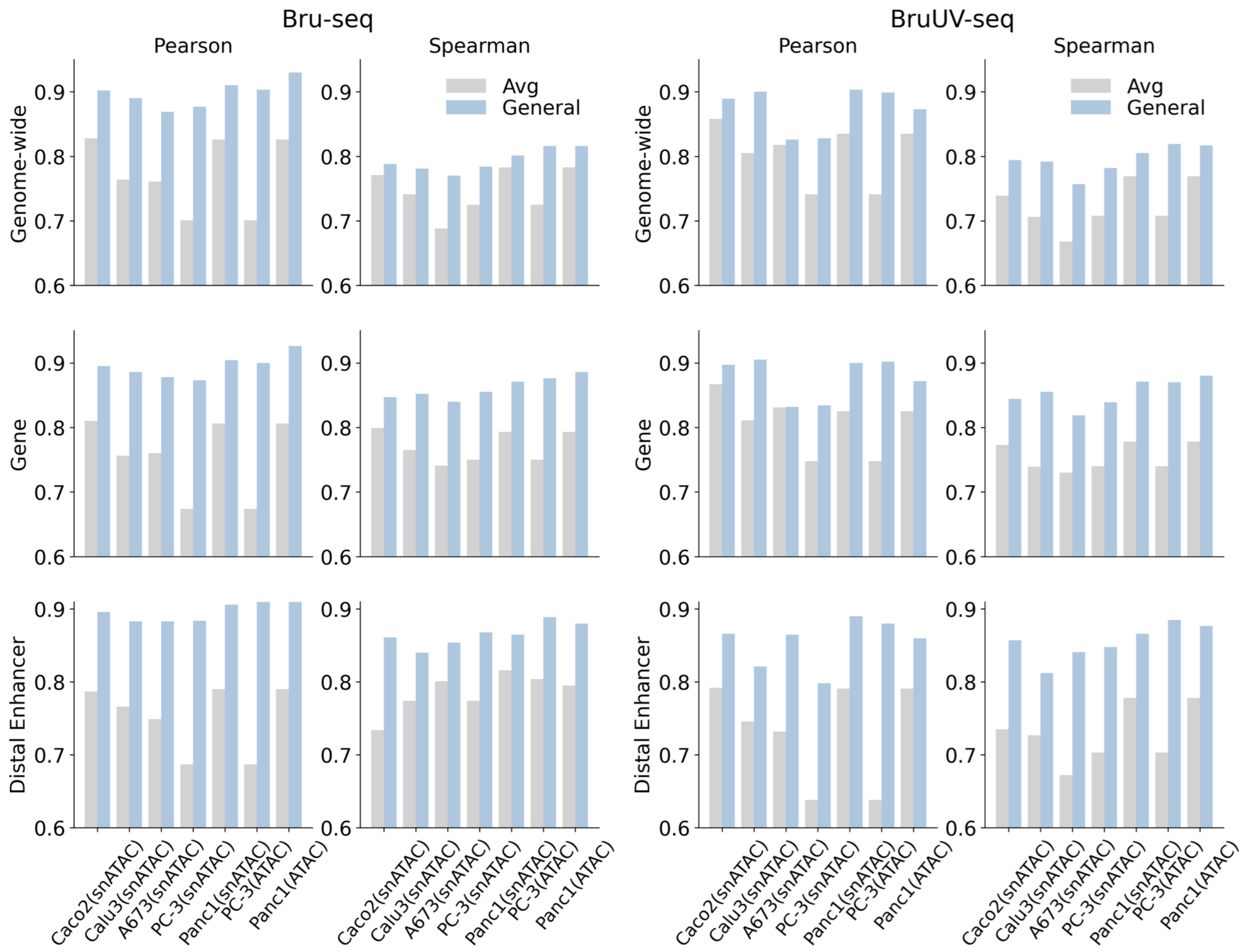

Fig. S7: Prediction performance comparisons between the average signal of all training cell lines and the general model using snATAC-seq or ATAC-seq on Bru-seq and BruUV-seq. The general model outperforms the average signal experiment across genomic bins associated with the whole genome, genes, and candidate distal enhancer elements in chromosomes 10 and 21. For PC-3 and Panc1 cell lines, we use both snATAC-seq and ATAC-seq, while for the other three cell lines, we use snATAC-seq.

Cluster	Cell number
HC	420
PC/DC	269
Roof	253
MES	107
Endo	83
Immune	78

(a) scATAC data statistics

Cluster	Cell number
HC	51
PC/DC	82
Roof	22
MES	150
Endo	71
Immune	18
latSC	39
medSC	31
PRO	34
SAT	146
SCH	51

(b) scRNA data statistics

Table S1: Statistics of scATAC data and scRNA data

bioRxiv preprint doi: https://doi.org/10.1101/2025.05.08.652986; this version posted May 14, 2025. The copyright holder for this preprint (which was not certified by peer review) is the author/funder, who has granted bioRxiv a license to display the preprint in perpetuity. It is made available under aCC-BY 4.0 International license.

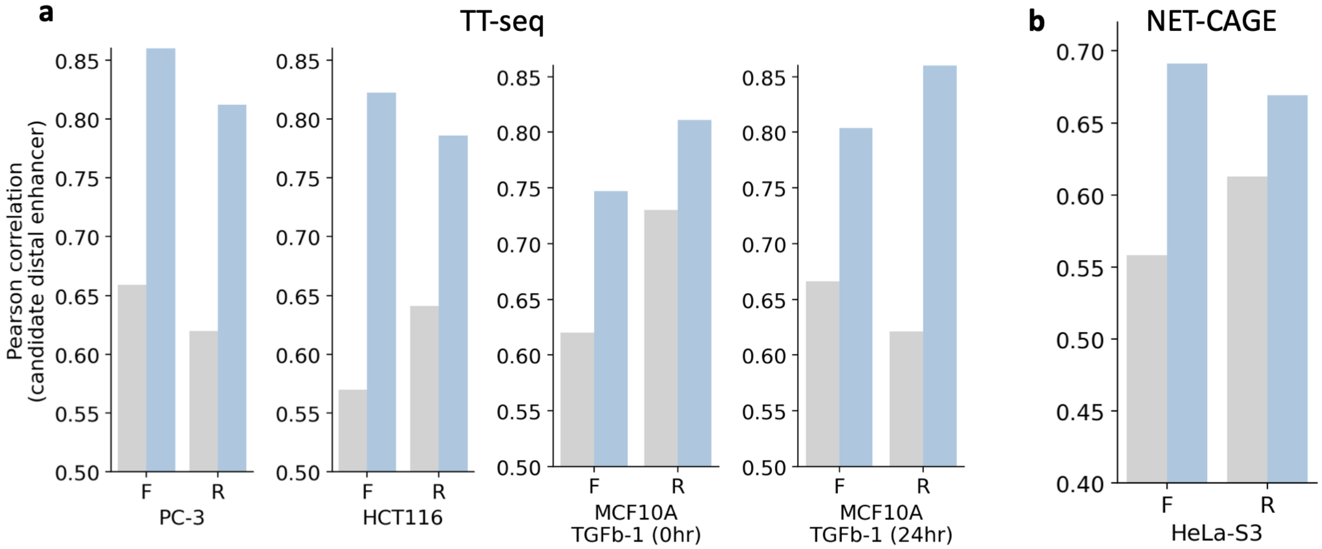

Fig. S8: Prediction performance on TT-seq (a) and NET-CAGE (b) across candidate enhancer elements. Our model makes predictions more accurately than average experimental signals.

bioRxiv preprint doi: https://doi.org/10.1101/2025.05.08.652986; this version posted May 14, 2025. The copyright holder for this preprint (which was not certified by peer review) is the author/funder, who has granted bioRxiv a license to display the preprint in perpetuity. It is made available under aCC-BY 4.0 International license.

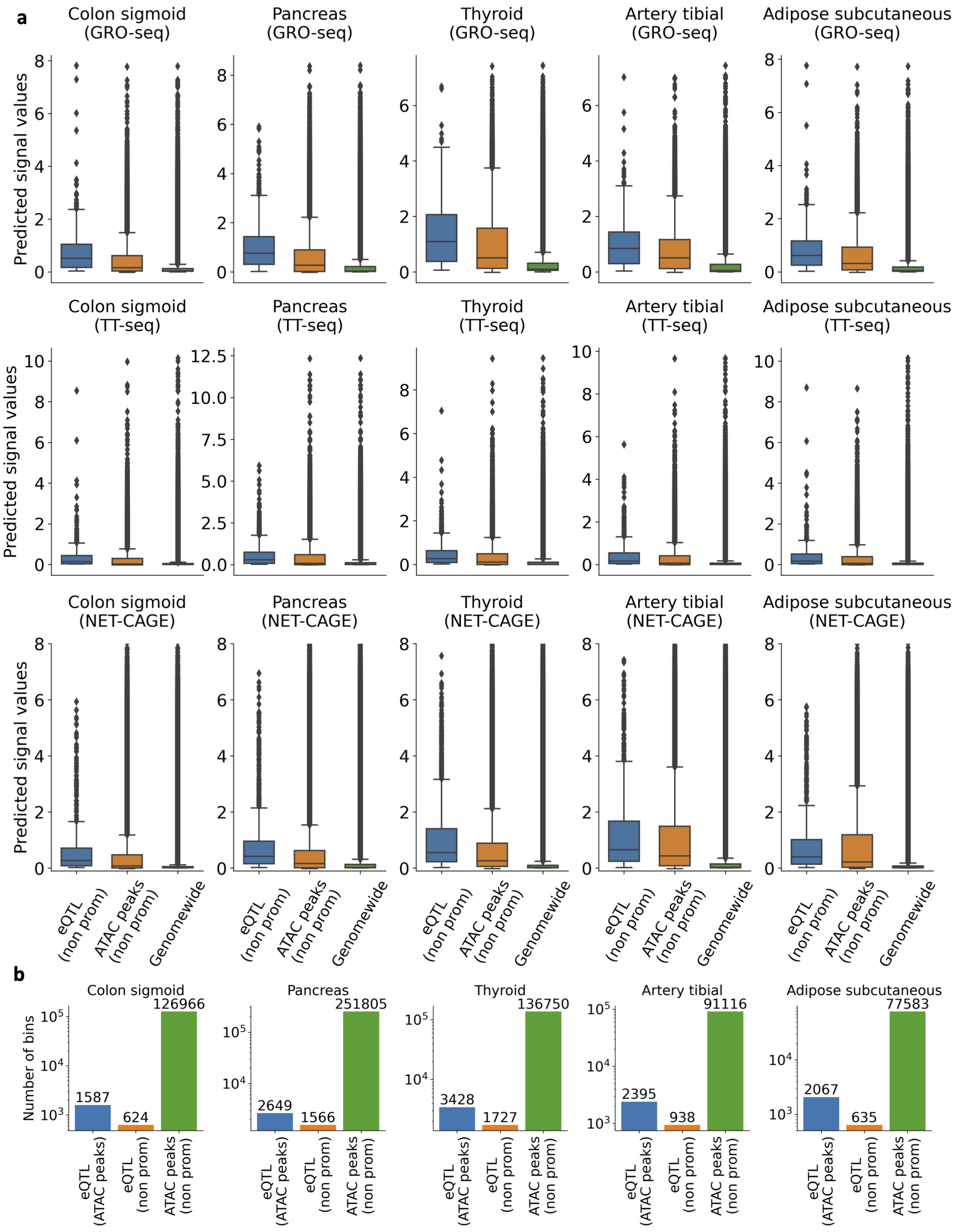

Fig. S9: See captions on the next page.

Fig. S9: Predictions of GRO-seq, TT-seq, and NET-CAGE on five tissues. **a**, The predicted signal values for three types of regions are displayed. The first type associates with genomic bins containing eQTLs that have a PIP score greater than 0.1 and overlap with corresponding non-promoter ATAC-seq peak regions. For comparative analysis, we also use genomic bins that overlap with non-promoter ATAC-seq peak regions and all genomic bins as a background comparison. Generally, the model predicts higher signal intensities at eQTL loci. **b**, The numbers of bins associated with eQTLs in ATAC-seq peaks, eQTLs in non-promoter ATAC-seq peaks, and non-promoter ATAC-seq peaks in five tissues.

bioRxiv preprint doi: https://doi.org/10.1101/2025.05.08.652986; this version posted May 14, 2025. The copyright holder for this preprint (which was not certified by peer review) is the author/funder, who has granted bioRxiv a license to display the preprint in perpetuity. It is made available under aCC-BY 4.0 International license.

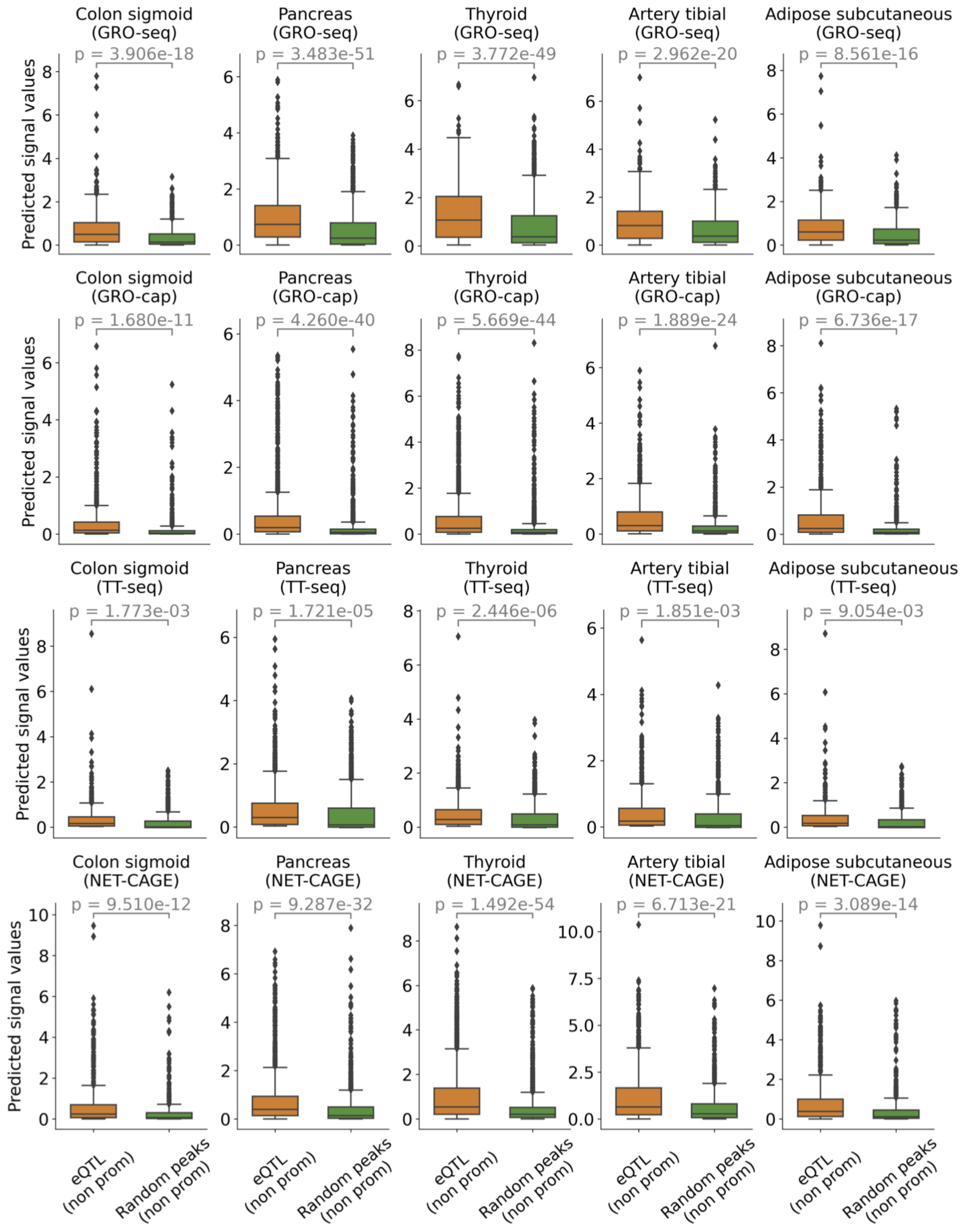

Fig. S10: The general model predicts higher signal values in eQTL regions that overlap with non-promoter open regions compared to those in non-promoter open regions only. We first obtain the predicted GRO-seq, GRO-cap, TT-seq, and NET-CAGE signal values for genomic bins associated with eQTLs (PIP > 0.1) located in non-promoter open regions. We then randomly sample an equivalent number of genomic bins exclusively from non-promoter open regions and record their signal values. Subsequently, we perform a t-test on these predicted values to determine whether the signal values in eQTL regions are significantly higher.

bioRxiv preprint doi: https://doi.org/10.1101/2025.05.08.652986; this version posted May 14, 2025. The copyright holder for this preprint (which was not certified by peer review) is the author/funder, who has granted bioRxiv a license to display the preprint in perpetuity. It is made available under aCC-BY 4.0 International license.

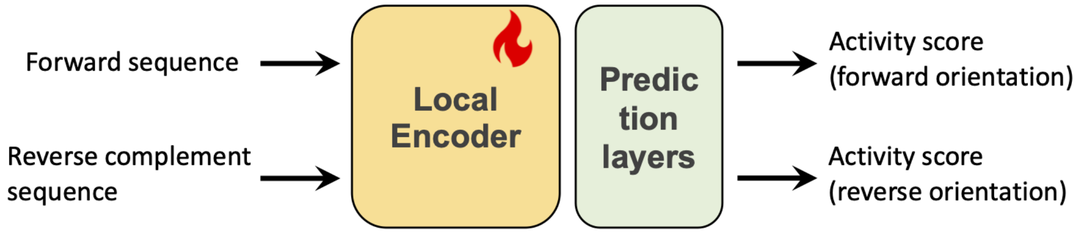

Fig. S11: Description of the LentiMPRA prediction task. We utilized both forward and reverse complement sequences to predict regulatory activity scores in corresponding orientations. This prediction is achieved by fine-tuning the local encoder and training additional convolutional and linear layers.

bioRxiv preprint doi: https://doi.org/10.1101/2025.05.08.652986; this version posted May 14, 2025. The copyright holder for this preprint (which was not certified by peer review) is the author/funder, who has granted bioRxiv a license to display the preprint in perpetuity. It is made available under aCC-BY 4.0 International license.

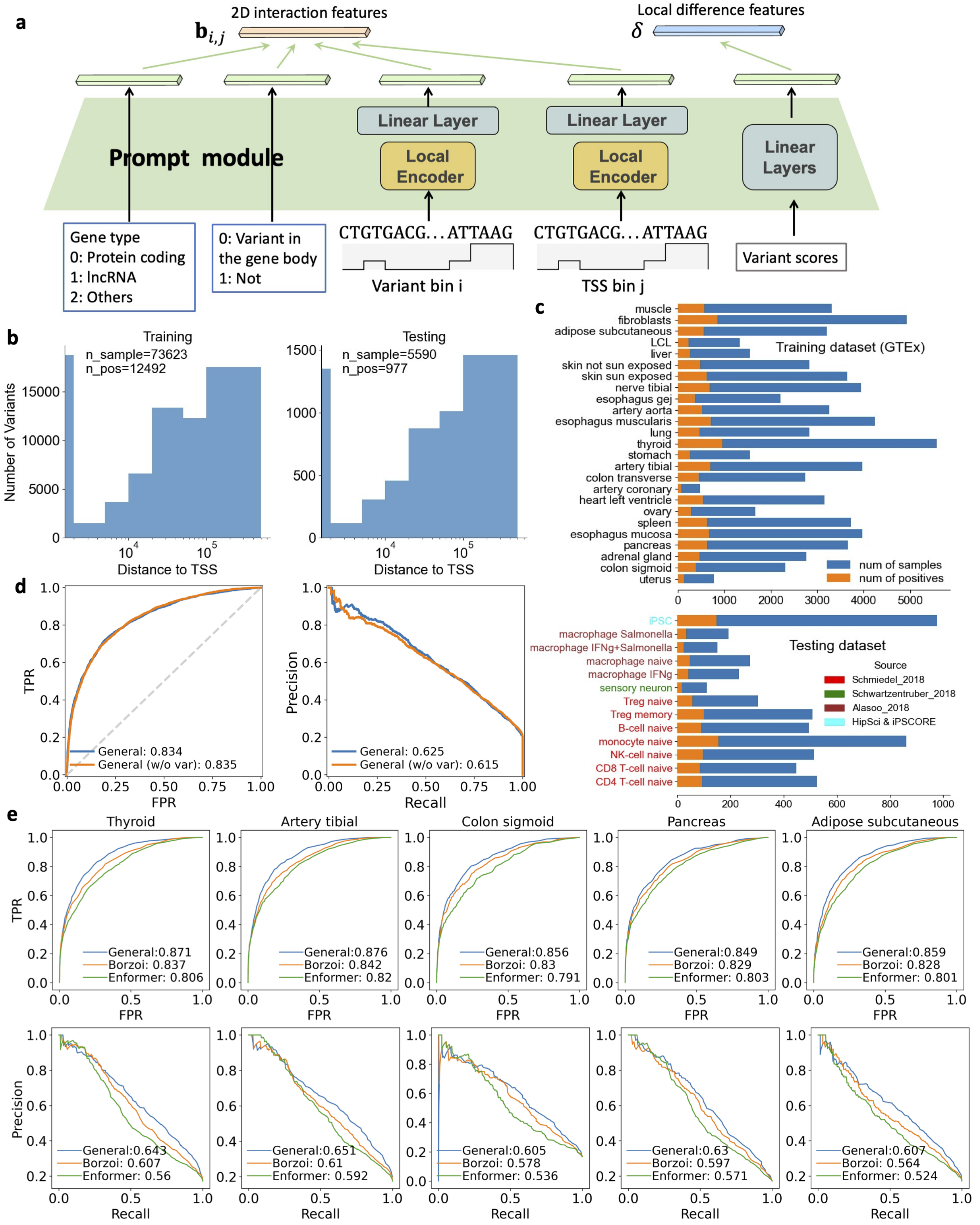

Fig. S12: See captions on the next page.

bioRxiv preprint doi: https://doi.org/10.1101/2025.05.08.652986; this version posted May 14, 2025. The copyright holder for this preprint (which was not certified by peer review) is the author/funder, who has granted bioRxiv a license to display the preprint in perpetuity. It is made available under aCC-BY 4.0 International license.

Fig. S12: Using the general model to improve eQTL classification. **a**, The architecture of the prompt module introduces extra information. First, gene type information, including protein-coding, lncRNA, and other gene types, along with the position of the variant (whether in the gene body), are converted into learnable embedding vectors. Then, these vectors and two instance-dependent vectors generated from the variant bin i and TSS bin j using the local encoder from the general model, are integrated into the 2D interaction feature $b_{i,j}$. Additionally, a Borzoi variant score vector is processed through linear layers and then incorporated into the local feature difference vector δ. **b**, Distribution of variants by distance to TSS in training and testing sets. The histograms show the number of variants as a function of their distance to the TSS. The number of samples, including those positive samples (causal eQTLs), is provided. **c** The number of samples, including the positive ones, in each cell or tissue from the training and testing datasets is provided. **d**, Our model achieves similar performance whether using Borzoi's variant scores as extra information in cross-cell type and study eQTL prediction. **e**, Integrating the representations of the general model with Borzoi variant scores outperforms both Borzoi and Enformer models in five tissues. The AUROC and PR curves are visualized.

bioRxiv preprint doi: https://doi.org/10.1101/2025.05.08.652986; this version posted May 14, 2025. The copyright holder for this preprint (which was not certified by peer review) is the author/funder, who has granted bioRxiv a license to display the preprint in perpetuity. It is made available under aCC-BY 4.0 International license.

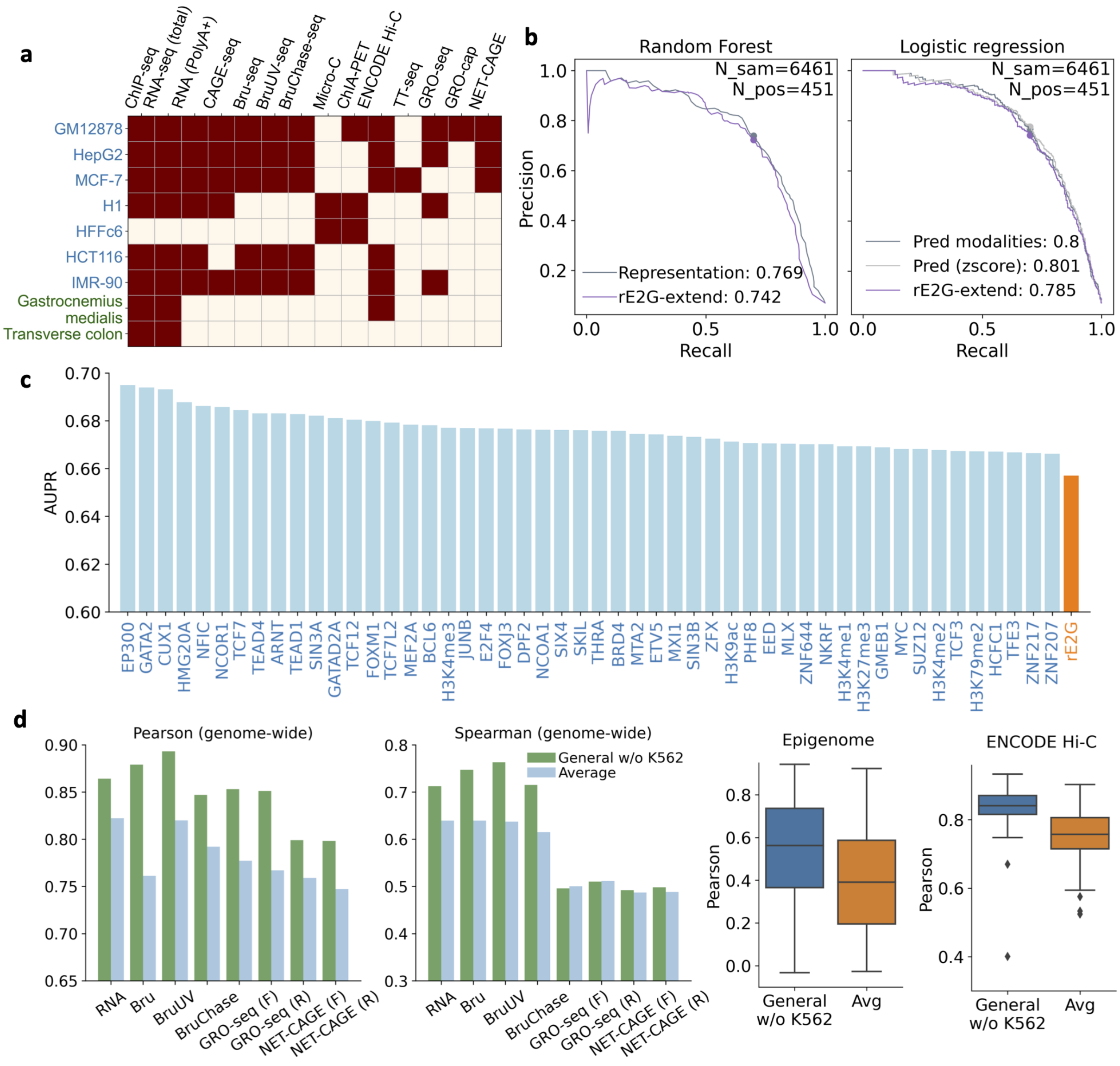

Fig. S13: Evaluation results on the K562 CRISPR perturbation dataset. **a**, The cells, tissues, and genomic modalities used to retrain the general model were selected to exclude the K562 cell line, thus preventing information leakage during testing on the perturbation dataset. **b**, AUPR scores resulting from the introduction of individual predicted TFs to the ENCODE rE2G features are presented. The top 20 TFs showing the greatest improvement are displayed, with the original ENCODE rE2G score provided for comparison. **c**, Both the representations and predicted modalities improve the ENCODE rE2G-extend model. A random forest algorithm is applied to the z-score normalized representations and features used in the ENCODE rE2G-extend model. Logistic regression is employed for the predicted modalities, both with and without z-score normalization, along with the ENCODE rE2G-extend features. The predicted modalities include RNA-seq, CAGE-seq, GRO-seq, GRO-cap, NET-CAGE, and O/E and KR normalized intact Hi-C and Micro-C contact maps. Note that predicted histone marks and EP300 are not included, as their experimental data has already been incorporated into the ENCODE rE2G-extend features. d, Prediction performance comparisons are shown between average signals using data from Figure 1e, excluding K562 data, and the general models using only K562 ATAC-seq. The general model consistently achieves better performance.

bioRxiv preprint doi: https://doi.org/10.1101/2025.05.08.652986; this version posted May 14, 2025. The copyright holder for this preprint (which was not certified by peer review) is the author/funder, who has granted bioRxiv a license to display the preprint in perpetuity. It is made available under aCC-BY 4.0 International license.

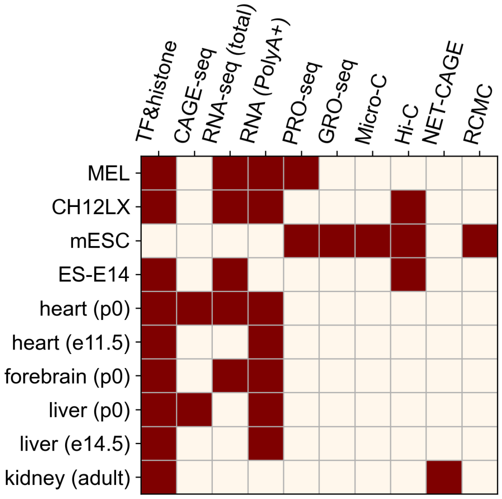

Fig. S14: Overview of the cells and tissues as well as the modalities used in the training of mouse general model.

bioRxiv preprint doi: https://doi.org/10.1101/2025.05.08.652986; this version posted May 14, 2025. The copyright holder for this preprint (which was not certified by peer review) is the author/funder, who has granted bioRxiv a license to display the preprint in perpetuity. It is made available under aCC-BY 4.0 International license.

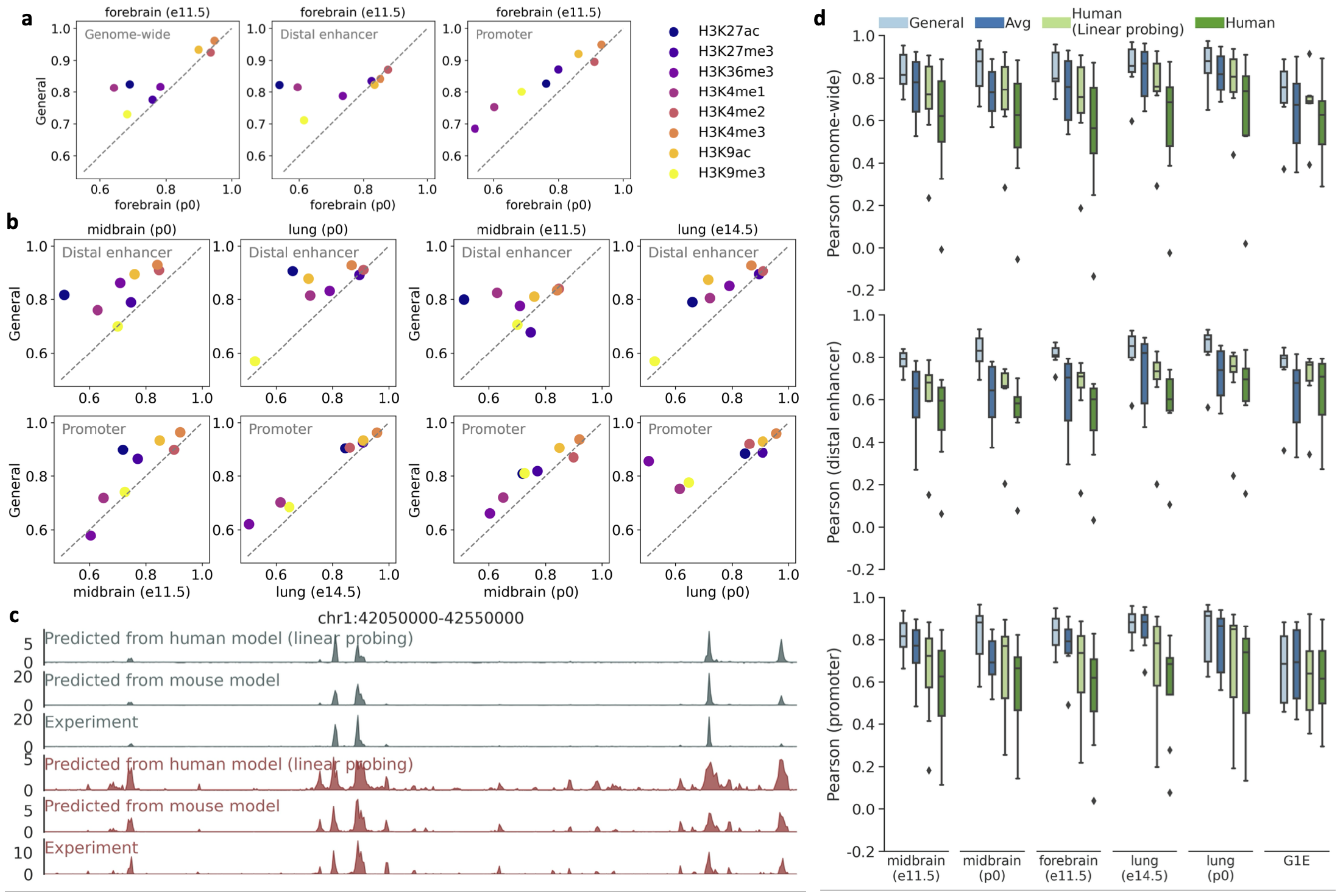

Fig. S15: Prediction performance on mouse histone mark prediction. **a**, The general model predicts histone marks in forebrain embryos more accurately than using the experimental histone marks from forebrain postnatal across the genome, distal enhancers, and promoter regions in chromosome 1. The Pearson correlation scores are reported. **b**, The general model more accurately predicts histone marks in the midbrain and lung at one developmental stage compared to using their experimental embryonic histones from another developmental stage, across distal enhancer and promoter regions. **c**, A region to show that mouse model predicts histone marks more accurately than the human model using the linear probing method. **d**, Comparison of prediction performance among the mouse general model, average signals of all training cells and tissues, and the human model with or without fine-tuning of the last linear layer. The mouse general model consistently achieves better performance across the genome, distal enhancer, and promoter regions in chromosome 1.

bioRxiv preprint doi: https://doi.org/10.1101/2025.05.08.652986; this version posted May 14, 2025. The copyright holder for this preprint (which was not certified by peer review) is the author/funder, who has granted bioRxiv a license to display the preprint in perpetuity. It is made available under aCC-BY 4.0 International license.

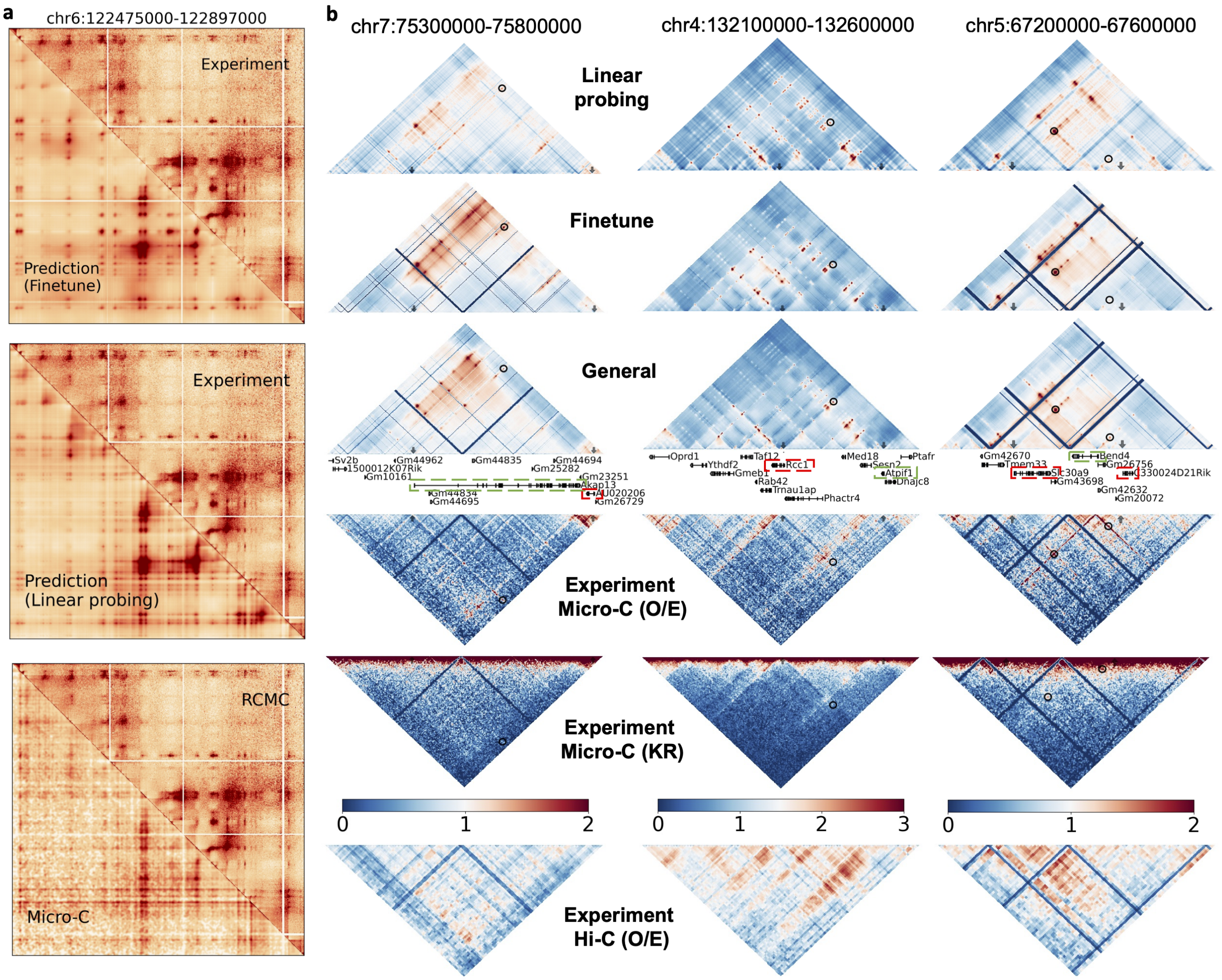

Fig. S16: Evaluations on the predicted RCMC contact maps. **a**, Comparison of the RCMC experimental data with predictions from the fine-tuning model, linear probing model, and experimental Micro-C. **b**, Comparison of the predictions of four regulatory interactions from a promoter knockout study [55] among three approaches. The general model identifies all four interactions, while the fine-tuning model identifies three. Although the linear probing approach also identifies four interactions, the patterns are not as clear as those predicted by the general model. For example, the interaction between Bend4 and C330024D21Rik is less distinct. The KR-normalized Micro-C only clearly identifies one interaction. Although the interactions have a large signal in O/E-normalized Micro-C, they do not all exhibit loop patterns, such as in the BEND4 interaction example. Furthermore, the Hi-C contact map fails to detect all these interactions.

bioRxiv preprint doi: https://doi.org/10.1101/2025.05.08.652986; this version posted May 14, 2025. The copyright holder for this preprint (which was not certified by peer review) is the author/funder, who has granted bioRxiv a license to display the preprint in perpetuity. It is made available under aCC-BY 4.0 International license.

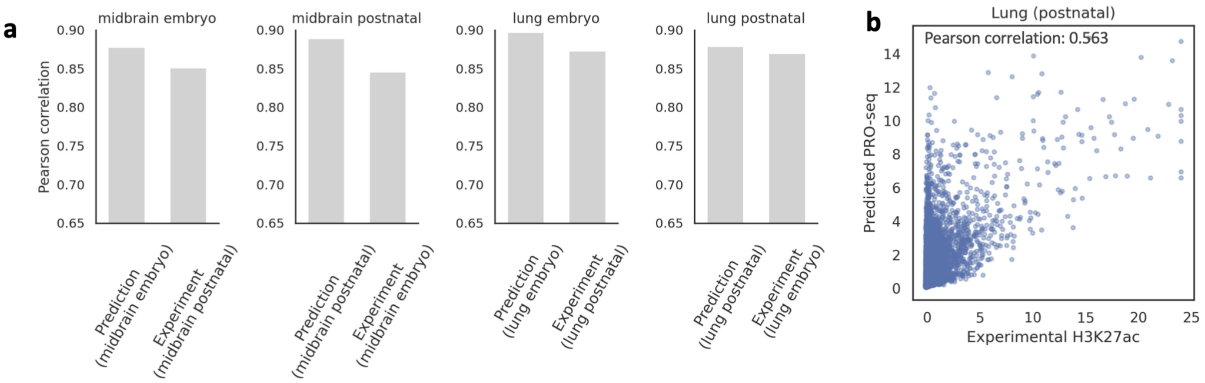

Fig. S17: Evaluation of predicted gene expression and enhancer RNA signals. **a**, Pearson correlation scores comparing predicted and experimental RNA-seq data for midbrain and lung tissues across embryonic and postnatal stages. For each developmental stage (e.g., embryo), predictions based on experimental data from the same stage show higher correlations compared to using experimental data from a different stage. **b**, Correlation between predicted PRO-seq signals and experimental H3K27ac data across candidate enhancer regions in chromosome 1 of postnatal lung tissue.

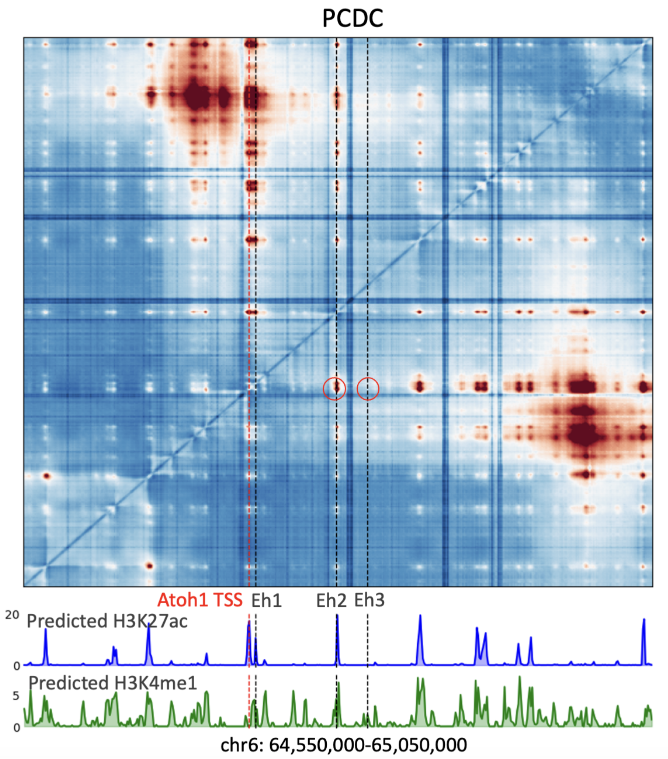

Fig. S18: The model predicts a silencer at the En3 locus in the PCDC cell type. This locus lacks a clear predicted loop with the Atoh1 TSS and shows no predicted H3K27ac or H3K4me1 peaks, consistent with its silencer function.
